# Association of Aging Trajectories in the Japan Science and Technology Agency Index of Competence With Instrumental Activities of Daily Living Among Community‐Dwelling Older Japanese Adults: The Otassha Study

**DOI:** 10.1111/ggi.70232

**Published:** 2025-10-21

**Authors:** Hisashi Kawai, Keigo Imamura, Manami Ejiri, Yoshinori Fujiwara, Kazushige Ihara, Hirohiko Hirano, Hiroyuki Sasai, Shuichi Obuchi, Takao Suzuki

**Affiliations:** ^1^ Tokyo Metropolitan Institute for Geriatrics and Gerontology Tokyo Japan; ^2^ Faculty of Medicine Hirosaki University Aomori Japan; ^3^ National Center for Geriatrics and Gerontology Aichi Japan

**Keywords:** group‐based trajectory modeling, higher‐level functional capacity, instrumental activities of daily living, Japan Science and Technology Agency Index of Competence, Tokyo Metropolitan Institute of Gerontology Index of Competence

## Abstract

**Aim:**

The Japan Science and Technology Agency Index of Competence (JST‐IC) was developed to measure competence at much higher levels than the Tokyo Metropolitan Institute of Gerontology Index of Competence (TMIG‐IC), which evaluates higher‐level functional capacity necessary for independent living. This study explored the JST‐IC aging trajectories and their associations with instrumental activities of daily living (IADL) impairment in community‐dwelling older Japanese individuals.

**Methods:**

Participants were 665 adults who responded to the 2014 baseline survey and at least one follow‐up survey (2015–2019) of the Otassha study. The JST‐IC and TMIG‐IC aging trajectories were identified using group‐based trajectory modeling. The association with nine‐year IADL impairment onset was investigated using Cox proportional hazards models.

**Results:**

Three JST‐IC trajectory groups were identified: high (29.5%), medium (53.1%), and low (17.3%), with scores decreasing by one to two points with increasing age over the follow‐up period. High‐stable (50.6%), late‐onset decrease (41.1%), and early‐onset decrease (8.3%) TMIG‐IC trajectory groups were identified with a one‐ to three‐point decrease in late‐ and early‐onset groups. The low JST‐IC trajectory group had an adjusted hazard ratio of 2.90 (95% confidence interval, 1.60–5.24) for IADL impairment (reference: high group). Low technology usage, information practices, and social engagement trajectories were significantly associated with IADL impairment.

**Conclusions:**

The JST‐IC scores gradually declined in all groups, even among those who maintained their TMIG‐IC scores. The low JST‐IC trajectory group—particularly low technology usage, information practices, and social engagement—was associated with the onset of IADL impairment.

## 1 Introduction

Globally, the population aged 65 years and above is anticipated to achieve the highest growth, reaching 11.7% in 2030 and 15.9% in 2050 [1]. This underscores the need to assess people's competence to live independently later in life. In Japan, the proportion of the population aged 65 years and above was 29.1% in 2023 [2], indicating a higher aging rate than in other countries.

Japan's need for an indicator to assess older adults' competence to live independently led to the development of the Tokyo Metropolitan Institute of Gerontology Index of Competence (TMIG‐IC) [3]. The TMIG‐IC is a 13‐item questionnaire that assesses the three higher levels of functional capacity defined by Lawton [4]: instrumental activities of daily living (IADL), intellectual activity, and social role. It has been used in numerous epidemiological studies to identify the characteristics of decline in higher‐level functional capacity among older adults [5, 6] and to clarify its impact on subsequent health outcomes as an indicator of IADL disability [7, 8, 9].

Over 30 years after the development of the TMIG‐IC, some recent studies have reported that the physical and cognitive functions of older Japanese individuals are improving; that is, they are “rejuvenating.” [10, 11, 12] The TMIG‐IC score has also demonstrated a remarkable improvement among older adults, both men and women, between 1988 and 1998, and again in 2008, suggesting significant improvement in the higher‐level functional capacity of community‐dwelling older Japanese people over the past 30 years [11]. This trend underscores the need for an indicator to evaluate a higher‐level functional capacity.

Consequently, the Japan Science and Technology Agency Index (JST‐IC) was developed to evaluate a higher functional capacity that could not be assessed using the TMIG‐IC [13]. It was developed considering increases in the following five aspects: (i) older adults living alone, (ii) demand for older individuals who perform productive activities, (iii) older victims of crime, (iv) older users of and advancements in electronic devices, and (v) usage of everyday life and health information by older individuals [14]. The index comprises 16 items and 4 subscales (technology usage, information practices, life management, and social engagement) necessary for life environment changes (Table S1) [13].

A previous study confirmed the factor structure, concurrent validity (with the TMIG‐IC, physical fitness, and health literacy), and internal consistency of the JST‐IC [13]. Several studies have used the JST‐IC to assess higher‐level functional capacity and activities of daily living in older Japanese individuals [15, 16, 17], and as an external validity criterion for indicators of new concepts, such as diversity in daily activities and social contact self‐efficacy [18, 19]. Regarding the characteristics of the JST‐IC, cohort studies have reported its association with reduced frequency of going out [20], cessation of driving [21], lower urinary tract symptoms [22], declines in physical and cognitive function [23], and cognitive frailty [24] in community‐dwelling older adults. Recent studies have examined the association between the technology usage subscale of the JST‐IC and frailty [25] and between the number of negative answers on the JST‐IC and the prevalence of physical and cognitive declines [26]. However, these studies were cross‐sectional; few longitudinal studies have used the JST‐IC to measure functional decline outcomes [27, 28]. Among the few longitudinal studies that used the JST‐IC as an outcome measure, they typically assessed it at only one or two time points. Consequently, no long‐term longitudinal studies have examined its association with health outcomes. As a result, both the longitudinal changes in JST‐IC scores and the association of JST‐IC scores with health outcomes remain unclear. Furthermore, no studies have compared the longitudinal changes in the JST‐IC with those in the TMIG‐IC. Investigating these aspects will contribute to a better understanding of how higher‐level functional capacity changes with age, and will help establish the predictive validity of the JST‐IC.

Therefore, this study aimed to investigate the longitudinal association of the JST‐IC aging trajectory patterns and the onset of IADL impairment in community‐dwelling older Japanese individuals, and to verify the predictive validity of the JST‐IC. In addition, we compared aging trajectory patterns of the JST‐IC with those of the TMIG‐IC.

## 2 Methods

### 2.1 Participants

**FIGURE 1:**
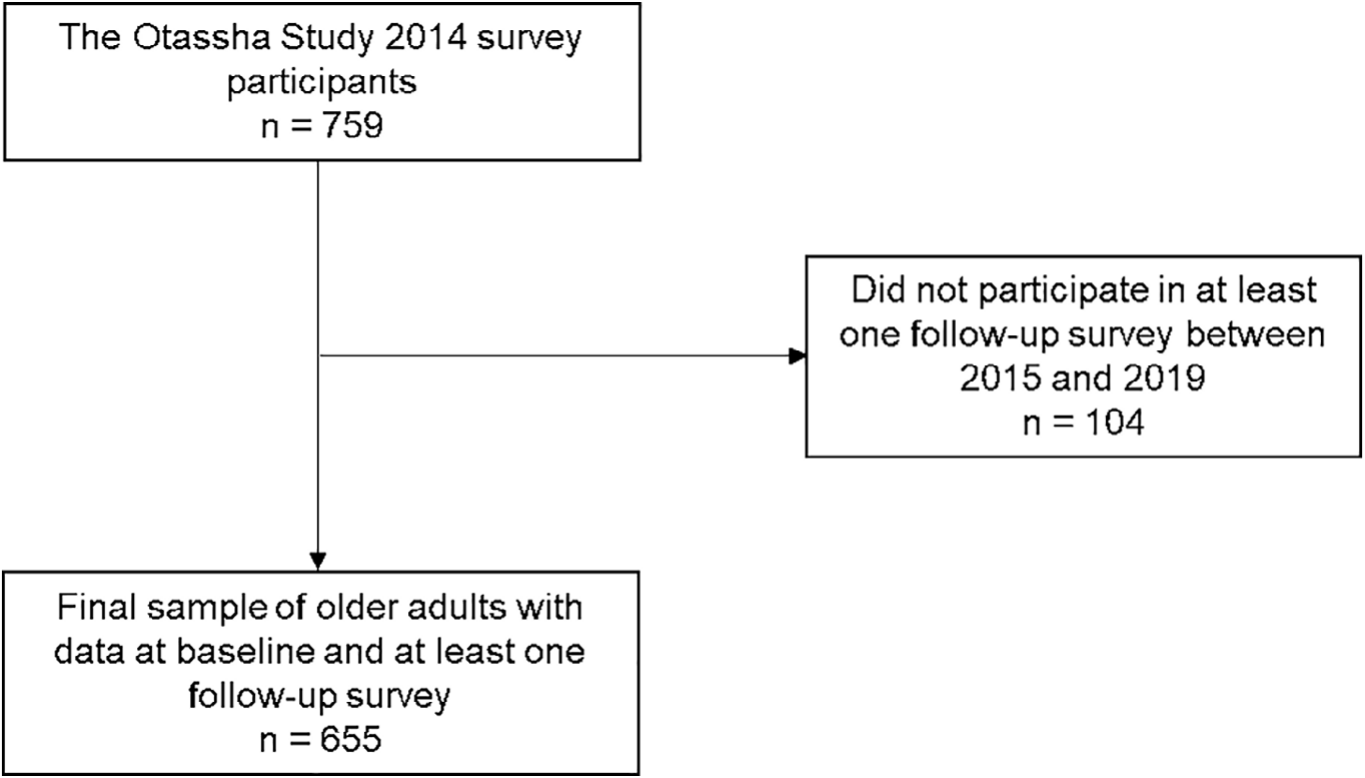
Flowchart of participants' selections.

The participants were community‐dwelling older adults who responded to the 2014 on‐site survey of “The Otassha Study 2011 cohort” and at least one of the follow‐up surveys between 2015 and 2019. Each annual survey was conducted in September and October. In the 2011 survey, the participants were recruited from approximately 7000 residents living in nine areas of Itabashi Ward, Tokyo, Japan (excluding those living in facilities and participants in our other cohort studies), and 913 participated in the on‐site survey. Annual follow‐up surveys were conducted, adding new 65‐year‐olds to the baseline participants each year [29, 30]. The data from 655 older adults who participated in the baseline survey in 2014 and at least one follow‐up were analyzed (Figure 1). The average number of surveys was 4.5 (standard deviation (SD) = 1.5), and the number of observations was 2967.

The participants provided written informed consent to use their data in this study. This study was approved by the Ethics Committee of the Tokyo Metropolitan Institute for Geriatrics and Gerontology (no. R22‐034) and conducted in accordance with the guidelines of the World Medical Association Declaration of Helsinki.

### 2.2 Assessment of the JST‐IC and TMIG‐IC

The JST‐IC and TMIG‐IC were assessed during interviews in annual on‐site surveys. We assessed the total JST‐IC score of 16 items and the following subscales: technology usage (Items 1–4), information practices (Items 5–8), life management (Items 9–12), and social engagement (Items 13–16) [13]. For the TMIG‐IC, we assessed the total score of 13 items and three subscales: IADL (Items 1–5), intellectual activity (Items 6–9), and social role (Items 10–13) [3]. In both the JST‐IC and TMIG‐IC, one point was added for “Yes” and zero for “No.” The JST‐IC scores ranged from 0 to 16 points, and the TMIG‐IC scores from 0 to 13 points, with higher scores indicating higher functional capacity.

### 2.3 IADL Impairment

IADL impairment was defined as a “no” on any of the five items of the IADL subscale of the TMIG‐IC (Table S2), assessing the competence to: go out, shop, eat, pay, and manage money. The JST‐IC expands the IADL to include device usage and productive activity [14], and uses different questions. The follow‐up status for IADL, such as independent, IADL impairment, and dropout, was assessed annually during the on‐site survey from 2014 to 2023. Those who already had IADL impairment in 2014 (*n* = 31) were excluded from the analysis. The status was recorded as “unknown” if a person did not participate in the survey. The months to determine the final follow‐up status were (1) October of the year when IADL impairment first occurred, (2) October of the year when there was no further year with the independent status, and the status first became “unknown,” or (3) October 2023.

### 2.4 Covariates

Age, gender, chronic diseases, and years of education at the time of the baseline survey in 2014 were assessed. The presence or absence of chronic diseases (hypertension, stroke, heart disease, and diabetes) was examined through interviews with nurses, and the total number of these diseases was calculated as the number of chronic diseases. Information on the total years of schooling was also obtained in the interviews.

### 2.5 Statistical Analysis

Aging trajectory patterns of the JST‐IC and TMIG‐IC total scores, as well as the JST‐IC subscale scores, from ages 65 to 85, were identified using group‐based trajectory modeling with the cnorm model in the TRAJ plugin for STATA 17.0 (StataCorp LLC, College Station, Texas, United States). Three trajectory patterns were identified in each score using a quadratic curve model. Based on our previous study [5] regarding the aging trajectory of TMIG‐IC, we approximated the trajectories using a quadratic curve model. For the total score, we examined models with four, three, and two groups, yielding Bayesian information criterion values of −5949.84, −6013.02, and −6233.35, respectively, with lower values observed for models with fewer groups. The entropy values were 0.762, 0.808, and 0.762, respectively, indicating that the three‐group model was best. The average of maximum posterior probability of assignment (APPA) and odds correction classification (OCC) [31] metrics exceeded the commonly accepted thresholds (APPA > 0.7 and OCC > 0.5); therefore, we adopted the three‐group model. For the subscales, trajectories were classified into three groups for mutual comparison, and the APPA and OCC generally met these criteria (Table S3). Among the JST‐IC total score trajectory groups, covariates, number of people included in subscale trajectory groups, and IADL impairment were compared using the Jonckheere–Terpstra test for continuous variables and the Mantel–Haenszel test for trend for categorical variables.

The association between aging trajectory groups of the JST‐IC total and subscale scores and IADL impairment was investigated using Cox proportional hazards models, adjusting for gender, age, number of chronic diseases, and years of education. In this study, based on the relaxed rule of 10 events per variable [32], we limited the number of explanatory variables to five, assuming five outcome events per predictor variable. In addition to gender and age, we included the number of chronic diseases (physical aspect) and the number of years of education (socioeconomic aspect) as covariates, as these factors are associated with the occurrence of IADL impairment. Participants with “unknown” status—due to dropouts from the survey or death—were treated as censored. However, because death may be a competing risk for IADL impairment, we conducted a sensitivity analysis using the Fine–Gray model, with death as a competing risk. The proportional hazards assumption was confirmed using the cumulative survival curves. SPSS version 27 (IBM Japan Ltd., Tokyo, Japan) was used for statistical analyses other than group‐based trajectory modeling.

In the trajectory modeling, missing values for the JST‐IC were assumed to be missing at random, and estimation proceeded accordingly. The mean number of measurements of JST‐IC was 4.52 (SD = 1.49). Among the covariates, only six cases had missing data for years of education; these were excluded from the regression analysis using listwise deletion. Given the small number of missing cases, their impact on the results is considered negligible.

## 3 Results

**FIGURE 2:**
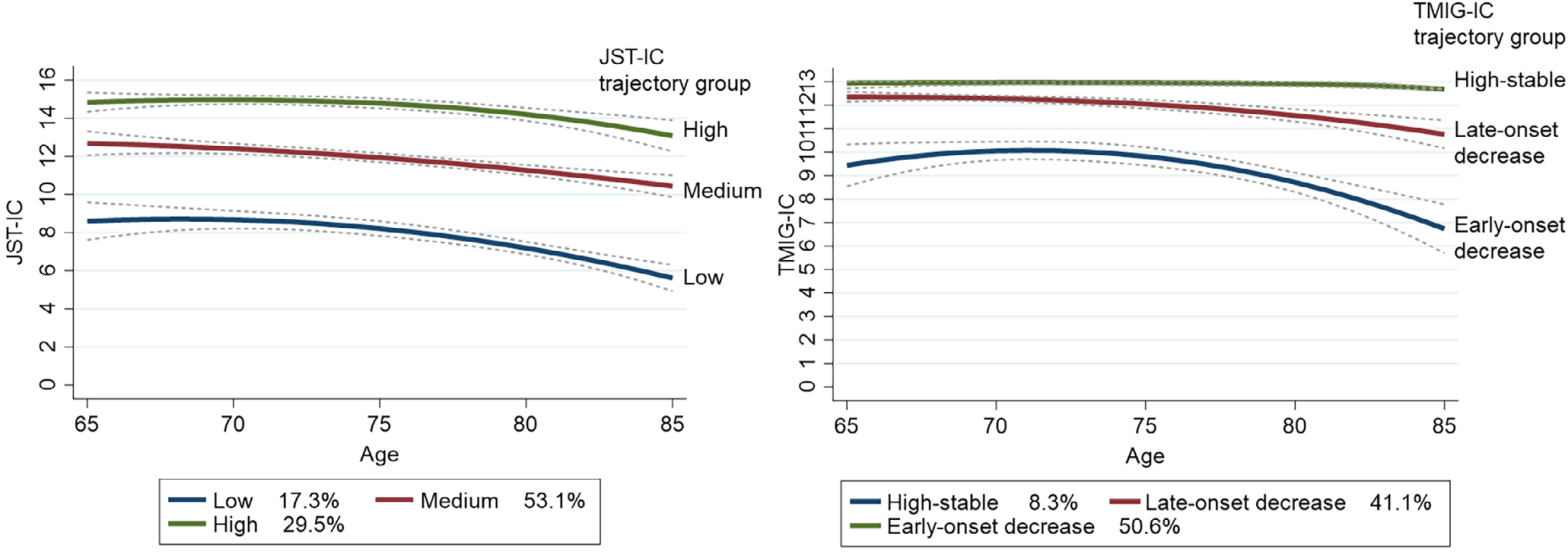
Comparison of aging trajectories between JST‐IC and TMIG‐IC in community‐dwelling older adults. Aging trajectories for the JST‐IC and TMIG‐IC were identified using a quadratic three‐group model through group‐based trajectory modeling. The optimal number of groups was determined based on the Bayesian information criterion, entropy, the average posterior probability of assignments, and the odds of correct classification for each trajectory. The reported percentages represent the proportion of participants assigned to each group. JST‐IC: Japan Science and Technology Agency Index of Competence; TMIG‐IC: Tokyo Metropolitan Institute of Gerontology Index of Competence.

Groups with high (29.5%), medium (53.1%), and low (17.3%) JST‐IC total score trajectories were identified, in which the scores decreased by one to two points with increasing age during follow‐up (Figure 2). High‐stable (50.6%), late‐onset decrease (41.1%), and early‐onset decrease (8.3%) TMIG‐IC trajectory groups were identified. The scores in late‐ and early‐onset decrease groups decreased by one to three points with increasing age during follow‐up, whereas the score remained almost unchanged in the high‐stable group.

**FIGURE 3:**
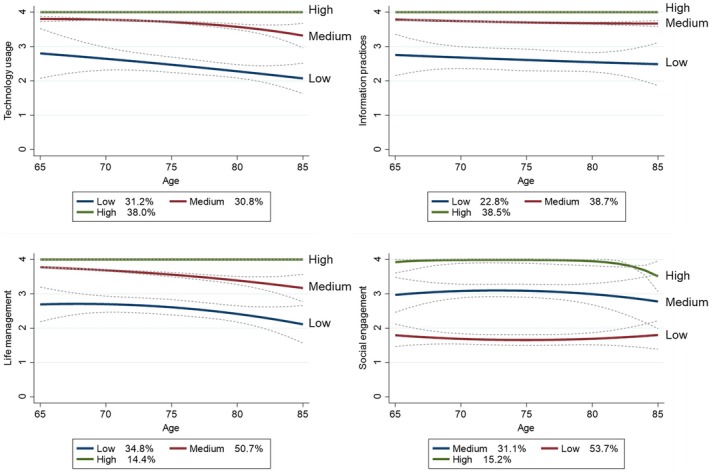
Aging trajectories of the JST‐IC subscales. Aging trajectories for the JST‐IC subscales were identified using a quadratic three‐group model through group‐based trajectory modeling. Each subscale was classified into three groups for mutual comparison. The average posterior probability of assignments and the odds of correct classification for each trajectory generally met the criterion. The reported percentages represent the proportion of participants assigned to each group. JST‐IC: Japan Science and Technology Agency Index of Competence.

The following groups were identified for technology usage and life management subscales: high with almost no decline, medium with a gradual decline from a high level, and low with a decline from the age of 65 years (Figure 3). The difference between the high and medium score groups in the information practices subscale was small, and the scores did not change over time. However, almost no decline was observed in the low information practices group. For social engagement, approximately one point difference was observed between the high, medium, and low groups in all age categories; however, the change over time was smaller than that in technology usage and life management.

Significant trends in the characteristics of the lower trajectory group were observed, including higher age, higher prevalence of hypertension and heart disease, greater number of chronic diseases, and fewer years of education compared to the high and medium JST‐IC total score trajectory groups (Table 1). The lower the total score, the more likely it belonged to the lower group of subscale scores. For the final follow‐up status in IADL, independence was significantly lower, and the follow‐up period was shorter in the lower total score group. Incidence of IADL impairment = 110 persons/(624 persons × 9 years) × 1000 persons = 19.6/1000 person‐years.

**TABLE 1:** Characteristics of the participants in each JST‐IC trajectory group.

Variable	JST‐IC trajectory group						*p* for trend^a^
	Low (*n* = 109)		Medium (*n* = 361)		High (*n* = 185)		
	*n*	%	*n*	%	*n*	%	
	Mean	SD	Mean	SD	Mean	SD	
Gender (women)	59	54.1%	220	60.9%	112	60.5%	0.357
Age (year)	74.8	5.54	73.3	5.65	73.1	5.42	**0.029**
Hypertension	57	52.3%	149	41.4%	72	38.9%	**0.038**
Stroke	12	11.0%	16	4.4%	9	4.9%	0.062
Heart disease	22	20.2%	51	14.2%	19	10.3%	**0.020**
Diabetes	13	11.9%	41	11.4%	16	8.6%	0.326
Number of chronic diseases (number)	1.0	0.84	0.7	0.81	0.6	0.78	**0.001**
Education (year)	11.9	2.48	12.9	2.76	13.4	2.67	< **0.001**
Technology usage trajectory group							
Low	79	72.5%	109	30.2%	11	5.9%	< **0.001**
Medium	14	12.8%	98	27.1%	53	28.6%	
High	16	14.7%	154	42.7%	121	65.4%	
Information practices trajectory group							
Low	80	73.4%	55	15.2%	7	3.8%	< **0.001**
Medium	23	21.1%	151	41.8%	51	27.6%	
High	6	5.5%	155	42.9%	127	68.6%	
Life management trajectory group							
Low	88	80.7%	117	32.4%	12	6.5%	< **0.001**
Medium	19	17.4%	201	55.7%	115	62.2%	
High	2	1.8%	43	11.9%	58	31.4%	
Social engagement trajectory group							
Low	105	96.3%	269	74.5%	18	9.7%	< **0.001**
Medium	2	1.8%	73	20.2%	96	51.9%	
High	2	1.8%	19	5.3%	71	38.4%	
Final follow‐up status for IADL							
Independent	25	26.0%	154	44.4%	101	55.8%	< **0.001**
IADL impairment	25	26.0%	63	18.2%	22	12.2%	
Dropout	46	47.9%	130	37.5%	58	32.0%	
Follow‐up month (month)	70.9	33.59	87.4	29.21	97.8	20.76	< **0.001**

*Note:* Bold values indicate a significant trend (*p* < 0.05).

Abbreviations: IADL: instrumental activities of daily living, JST‐IC: Japan Science and Technology Agency Index of Competence.

^a^ Continuous variables: Jonckheere–Terpstra test; categorical variables: Mantel–Haenszel test for trend.

The adjusted hazard ratios of the JST‐IC total score trajectory group for the nine‐year occurrence of IADL impairment were 1.74 (95% confidence interval: 1.07–2.84) and 2.90 (1.60–5.24) for the medium and low groups (reference: high group), respectively (Table 2). Adjusted hazard ratios of subscale trajectory groups significantly associated with IADL impairment were as follows: 2.19 (1.37–3.50) for low technology usage; 2.85 (1.66–4.88) and 2.62 (1.63–4.20) for low and medium information practices, respectively; and 2.44 (1.07–5.56) and 2.72 (1.25–5.92) for low and medium social engagement, respectively. Similar results were obtained in the Fine–Gray models with death as a competing risk (Table S4).

**TABLE 2:** Associations of the JST‐IC aging trajectory groups and the nine‐year onset of IADL impairment.

Trajectory group	Onset of IADL impairment			
	Adjusted hazard ratio	95% confidence interval		*p* ^a^
JST‐IC				
High	1			
Low	2.90	1.60	5.24	**< 0.001**
Medium	1.74	1.07	2.84	**0.026**
Technology usage				
High	1			
Low	2.19	1.37	3.50	**0.001**
Medium	1.05	0.62	1.76	0.868
Information practices				
High	1			
Low	2.85	1.66	4.88	< **0.001**
Medium	2.62	1.63	4.20	< **0.001**
Life management				
High	1			
Low	1.37	0.74	2.53	0.322
Medium	0.95	0.52	1.72	0.864
Social engagement				
High	1			
Low	2.44	1.07	5.56	**0.034**
Medium	2.72	1.25	5.92	**0.012**

*Note:* Bold values indicate a significant trend (*p* < 0.05).

Abbreviations: IADL: instrumental activities of daily living, JST‐IC: Japan Science and Technology Agency Index of Competence.

^a^ Cox proportional hazards models adjusting for gender, age, number of chronic diseases, and number of education years.

## 4 Discussion

This study aimed to clarify the characteristics of aging trajectory patterns using the JST‐IC, a new indicator of higher‐level functional capacity for independent living in older adults, and its predictive validity for health outcomes.

### 4.1 Characteristics of Aging Trajectory Patterns in the JST‐IC

Considering that the JST‐IC was developed to assess a higher functional ability than the TMIG‐IC, we attempted to capture the characteristics of the aging trajectory pattern of the JST‐IC through comparison with the TMIG‐IC. As the respondents could participate in the on‐site surveys of the cohort study, they most likely maintained their functional capacity. The high‐stable TMIG‐IC group accounted for 50.6% of the sample, higher than that in a previous study (36.3%) [6]. However, even among the participants of the present study, the JST‐IC declined over time in all groups; the JST‐IC can detect changes in higher‐level functional capacity that the TMIG‐IC cannot. The histogram of the TMIG‐IC indicated a ceiling effect at 13 points, whereas the JST‐IC showed a broader distribution range than the TMIG‐IC (Figure S1).

### 4.2 Aging Trajectory Patterns in the JST‐IC Subscales and Interpretations

In this study, we investigated the aging trajectory of each subscale to preliminarily understand which subscales are associated with the longitudinal decline in the JST‐IC total score and the occurrence of IADL impairment. The medium and low trajectory groups in technology usage and life management showed a decline over time, suggesting that the functions assessed by these subscales may decline in old age. However, changes in information practices and social engagement were stable, although they differed between the groups. In the JST‐IC, technology usage aimed to expand the scope of the TMIG‐IC's IADLs to include device usage, while life management aimed to expand the scope of intellectual activity and social roles to include the concepts of creativity and social interaction [14]. Several studies have suggested associations between age and information and communication technology (ICT) use [33], creativity [34], and social interaction [35, 36]; these factors may have influenced the declining aging trajectory pattern.

The information practices items included content regarding a positive attitude toward new information and the ability to gather necessary information, which may be related to high health literacy. While the association between low health literacy and the decline in physical and psychosocial factors associated with aging has been suggested in previous literature [37], the causal relationship remains unclear. In this study, information practices were maintained over time even in later life, when physical and psychosocial functions typically decline. As the study participants did not show significant deterioration in physical and psychosocial functions, it is possible that health literacy remained stable, contributing to the maintenance of information practices. However, because the analysis only included participants who continued throughout the longitudinal study, selection bias may be present. A decline in information practices may occur among more frail older adults over time. Social engagement includes items about participation in social activities. Physical and psychosocial factors may lead individuals to discontinue participating in social activities. Nevertheless, changes in social engagement were not remarkable in this study. However, this result may also be subject to selection bias.

### 4.3 Association Between JST‐IC Scores and the Onset of IADL Impairment

Compared with the high JST‐IC total score group, the medium and low groups had 1.7 and 2.9 times higher nine‐year risk of IADL impairment onset, respectively, indicating that the JST‐IC can predict the onset of IADL impairment. Regarding the subscales, low technology usage, medium and low information practices, and medium and low social engagement were associated with the onset of IADL impairment. A previous study [25], which defined ICT use using technology usage items on the JST‐IC, reported that ICT use is associated with physical frailty. Thus, technology usage can predict IADL impairment.

In this study, information practices and social engagement did not significantly decline over time. However, differences between the groups were evident in middle‐aged groups, suggesting that interventions, such as providing new information, education to improve information literacy, and promoting social participation for those with middle or low scores at age 65 years, are important to increase the number of older adults who can live independently.

### 4.4 Limitations

This study was conducted in an urban area in Japan, and data were collected from participants in consecutive cohort studies, thus limiting the generalizability of the results. Half the participants were healthy older adults who maintained their TMIG‐IC over time; therefore, the association with the risk of IADL impairment may have been underestimated in this study. The JST‐IC and TMIG‐IC scores in this sample may be lower than those in other populations, including those in rural areas; therefore, this study did not investigate the cut‐off value for IADL impairment. Future research should explore cut‐off values and evaluate discriminability for IADL impairment. Since this study used the rule of five outcome events per predictor variable, we were unable to adjust for sufficient covariates due to sample size limitations. Possible confounding factors, such as cognitive function, depression, and other socioeconomic conditions, should be considered in future research.

As the JST‐IC is a newly developed indicator with insufficient longitudinal data, this study analyzed data from one cohort, resulting in an inadequate sample size. Future studies should analyze longitudinal data from an integrated cohort study using JST‐IC data [26] and investigate the association with longer‐term outcomes, mortality, and hospitalization. Since the JST‐IC was developed with expanding IADL to higher levels, it was somewhat expected that it would be associated with IADL impairment. Therefore, future studies are necessary to examine the association with other geriatric outcomes, such as depression or cognitive function.

## 5 Conclusions

This study identified the aging trajectory patterns from the JST‐IC and examined their associations with IADL impairment onset in community‐dwelling older Japanese individuals. The results indicated that the JST‐IC assesses higher functional capacity than the TMIG‐IC and has predictive validity for the onset of IADL impairment.

## Conflicts of Interest

The authors declare no conflicts of interest.

## Supporting information


**Figure S1:** ggi70232‐sup‐0001‐FigureS1.docx.

**Table S1:** ggi70232‐sup‐0002‐TableS1.docx.

**Table S2:** ggi70232‐sup‐0003‐TableS2.docx.

**Table S3:** ggi70232‐sup‐0004‐TableS3.docx.

**Table S4:** ggi70232‐sup‐0005‐TableS4.docx.

## Data Availability

The data from the Otassha Study contains sensitive participant information and could not be publicly released because of the ethical and legal restrictions imposed by the Ethics Committee of the Tokyo Metropolitan Institute for Geriatrics and Gerontology.
