# Supplementary material for: Association of Aging Trajectories in the Japan Science and Technology Agency Index of Competence With Instrumental Activities of Daily Living Among Community‐Dwelling Older Japanese Adults: The Otassha Study
Source: Geriatr Gerontol Int. 2025 Oct 21;25(12):1894–902. doi: 10.1111/ggi.70232 (PMC12719133; doi:10.1111/ggi.70232)
Supplement: Supplementary file 1 — Figure S1: ggi70232‐sup‐0001‐FigureS1.docx. [file GGI-25-1894-s004.docx]

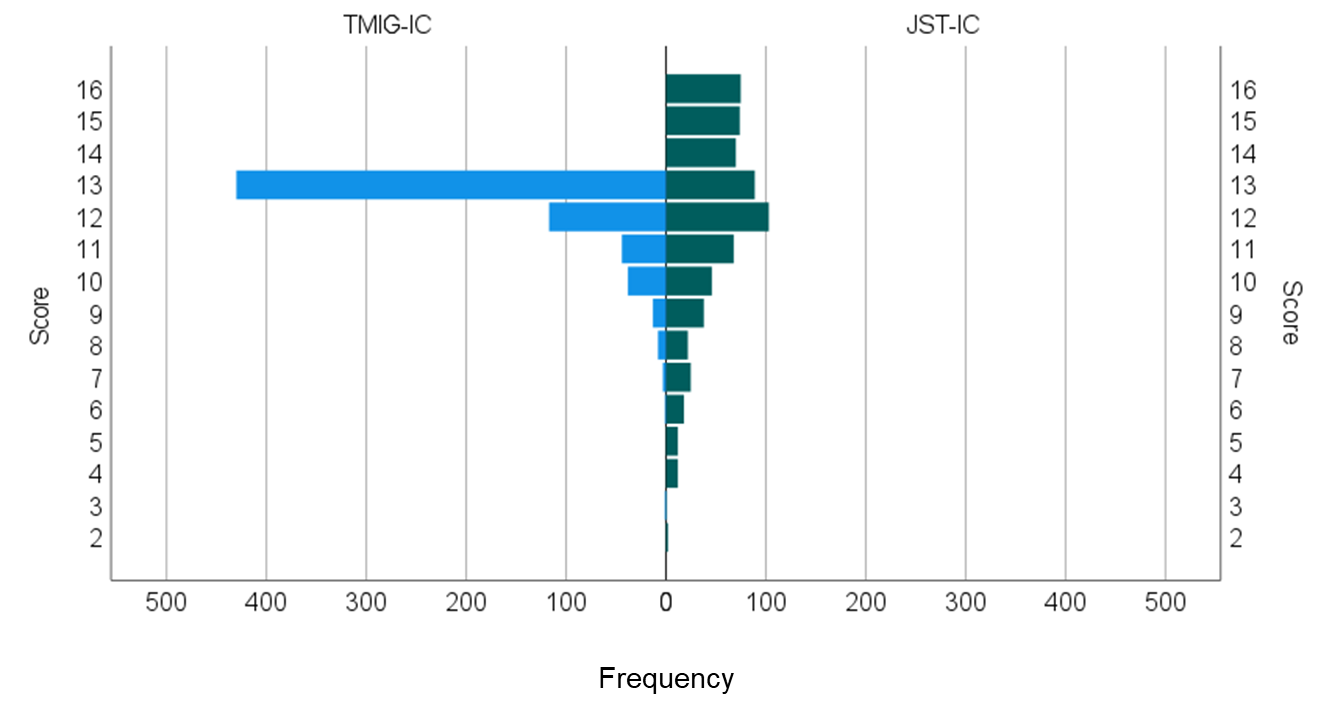


**Supplementary Figure 1.** Histograms of the JST-IC and TMIG-IC total scores in the 2014 survey among the final sample (n = 655).

The TMIG-IC has a ceiling effect at 13 points, while the JST-IC is distributed in a wider range than the TMIG-IC.

JST-IC: Japan Science and Technology Agency Index of Competence; TMIG-IC: Tokyo Metropolitan Institute of Gerontology
