# Supplementary material for: Association of Aging Trajectories in the Japan Science and Technology Agency Index of Competence With Instrumental Activities of Daily Living Among Community‐Dwelling Older Japanese Adults: The Otassha Study
Source: Geriatr Gerontol Int. 2025 Oct 21;25(12):1894–902. doi: 10.1111/ggi.70232 (PMC12719133; doi:10.1111/ggi.70232)
Supplement: Supplementary file 2 — Table S1: ggi70232‐sup‐0002‐TableS1.docx. [file GGI-25-1894-s001.docx]

**Supplementary Table 1.** Items and subscales of JST-IC.

| Subscale | Item | Score | |
| --- | --- | --- | --- |
| Technology usage | (1) Can you use a mobile phone? | Yes=1 | No=0 |
|  | (2) Can you use the ATM? | Yes=1 | No=0 |
|  | (3) Can you operate a video recorder such as a Blu-ray recorder or DVD player? | Yes=1 | No=0 |
|  | (4) Can you send an e-mail using a mobile phone or computer? | Yes=1 | No=0 |
| Information practices | (5) Are you interested in news and events from overseas? | Yes=1 | No=0 |
|  | (6) Can you determine the credibility of health-related information? | Yes=1 | No=0 |
|  | (7) Do you enjoy art, films, or music? | Yes=1 | No=0 |
|  | (8) Do you watch educational/cultural programs? | Yes=1 | No=0 |
| Life management | (9) Do you follow any measures to prevent yourself from becoming a victim of crimes. | Yes=1 | No=0 |
|  | (10) Do you try to be creative while doing daily tasks (i.e., cleaning, cooking)? | Yes=1 | No=0 |
|  | (11) Can you take care of an ill person? | Yes=1 | No=0 |
|  | (12) Do you take care of your grandchildren, family members, or acquaintances? | Yes=1 | No=0 |
| Social engagement | (13) Do you participate in regional festivals or events? | Yes=1 | No=0 |
|  | (14) Do you participate in a neighborhood association or a residents’ association? | Yes=1 | No=0 |
|  | (15) Would you be able to assume a managerial position such as an organizer in a residents’ association or group activities? | Yes=1 | No=0 |
|  | (16) Do you engage in charity or volunteer activities? | Yes=1 | No=0 |

JST-IC: Japan Science and Technology Agency Index of Competence
