# Supplementary material for: Association of Aging Trajectories in the Japan Science and Technology Agency Index of Competence With Instrumental Activities of Daily Living Among Community‐Dwelling Older Japanese Adults: The Otassha Study
Source: Geriatr Gerontol Int. 2025 Oct 21;25(12):1894–902. doi: 10.1111/ggi.70232 (PMC12719133; doi:10.1111/ggi.70232)
Supplement: Supplementary file 3 — Table S2: ggi70232‐sup‐0003‐TableS2.docx. [file GGI-25-1894-s002.docx]

**Supplementary Table 2.** Items of the IADL subscale of the TMIG-IC.

| Questionnaires | Score | |
| --- | --- | --- |
| 1. Can you use public transportation (bus or train) by yourself? | Yes=1 | No=0 |
| 2. Are you able to shop for daily necessities? | Yes=1 | No=0 |
| 3. Are you able to prepare meals by yourself? | Yes=1 | No=0 |
| 4. Are you able to pay bills? | Yes=1 | No=0 |
| 5. Can you handle your own banking? | Yes=1 | No=0 |
| TMIG-IC: Tokyo Metropolitan Institute of Gerontology Index of Competence, IADL: instrumental activities of daily living | | |
