# Supplementary material for: Association of Aging Trajectories in the Japan Science and Technology Agency Index of Competence With Instrumental Activities of Daily Living Among Community‐Dwelling Older Japanese Adults: The Otassha Study
Source: Geriatr Gerontol Int. 2025 Oct 21;25(12):1894–902. doi: 10.1111/ggi.70232 (PMC12719133; doi:10.1111/ggi.70232)
Supplement: Supplementary file 4 — Table S3: ggi70232‐sup‐0004‐TableS3.docx. [file GGI-25-1894-s003.docx]

**Supplementary Table 3.** Average posterior probabilities and odds of correct classification in the accepted trajectory groups.

| Trajectory group | | Average posterior probabilities | Odds of correct classification |
| --- | --- | --- | --- |
| JST-IC |  |  |  |
|  | Low | 0.936 | 73.626 |
|  | Medium | 0.903 | 7.545 |
|  | High | 0.917 | 28.183 |
| Technology usage | |  |  |
|  | Low | 0.905 | 22.079 |
|  | Medium | 0.889 | 24.958 |
|  | High | 0.865 | 7.691 |
| Information practices | |  |  |
|  | Low | 0.889 | 28.961 |
|  | Medium | 0.876 | 13.483 |
|  | High | 0.857 | 7.659 |
| Life management | |  |  |
|  | Low | 0.863 | 12.664 |
|  | Medium | 0.843 | 5.140 |
|  | High | 0.804 | 21.954 |
| Social engagement | |  |  |
|  | Low | 0.841 | 3.561 |
|  | Medium | 0.856 | 16.789 |
|  | High | 0.910 | 61.753 |
| JST-IC: Japan Science and Technology Agency Index of Competence | | | |
