# Supplementary material for: Association of Aging Trajectories in the Japan Science and Technology Agency Index of Competence With Instrumental Activities of Daily Living Among Community‐Dwelling Older Japanese Adults: The Otassha Study
Source: Geriatr Gerontol Int. 2025 Oct 21;25(12):1894–902. doi: 10.1111/ggi.70232 (PMC12719133; doi:10.1111/ggi.70232)
Supplement: Supplementary file 5 — Table S4: ggi70232‐sup‐0005‐TableS4.docx. [file GGI-25-1894-s005.docx]

**Supplementary Table 4.** Associations of the aging trajectory pattern groups of the JST-IC and the onset of IADL impairment for nine years, with death as a competing risk.

| Trajectory group | Onset of IADL impairment | | | |
| --- | --- | --- | --- | --- |
|  | Sub hazard ratio | 95% confidence interval | | P^*^ |
| JST-IC trajectory group | | |  |  |
| High | 1 |  |  |  |
| Low | 2.73 | 1.50 | 4.94 | **0.001** |
| Medium | 1.70 | 1.05 | 2.75 | **0.032** |
| Technology usage trajectory group | |  |  |  |
| High | 1 |  |  |  |
| Low | 2.12 | 1.28 | 3.49 | **0.003** |
| Medium | 1.05 | 0.62 | 1.79 | 0.855 |
| Information practices trajectory group | | |  |  |
| High | 1 |  |  |  |
| Low | 2.61 | 1.51 | 4.51 | **0.001** |
| Medium | 2.60 | 1.64 | 4.14 | **<0.001** |
| Life management trajectory group | | |  |  |
| High | 1 |  |  |  |
| Low | 1.31 | 0.71 | 2.43 | 0.384 |
| Medium | 0.92 | 0.51 | 1.64 | 0.766 |
| Social engagement trajectory group | | |  |  |
| High | 1 |  |  |  |
| Low | 2.66 | 1.21 | 5.85 | **0.015** |
| Medium | 2.44 | 1.08 | 5.53 | **0.032** |
| JST-IC: Japan Science and Technology Agency Index of Competence, IADL: instrumental activities of daily living | | | | |
| *Fine & Gray models adjusting for gender, age, number of chronic diseases, and education years. Bold values indicate a significant association. | | | | |
